# Extracellular matrix collagen I promotes the tumor progression of residual hepatocellular carcinoma after heat treatment

**DOI:** 10.1186/s12885-018-4820-9

**Published:** 2018-09-18

**Authors:** Rui Zhang, Min Ma, Xia-Hui Lin, Hua-Hua Liu, Jie Chen, Jun Chen, Dong-Mei Gao, Jie-Feng Cui, Zheng-Gang Ren, Rong-Xin Chen

**Affiliations:** Liver Cancer Institute, Zhongshan Hospital, Fudan University and Key Laboratory of Carcinogenesis and Cancer Invasion, Ministry of Education, Shanghai, China

**Keywords:** Hepatocellular carcinoma, Collagen I, ERK, Heat treatment

## Abstract

**Background:**

Accelerated malignant behaviors induced by insufficient thermal ablation have been increasingly reported, however, the exact mechanisms are still unclear. Here, we investigated the importance of the extracellular matrix (ECM) in modulating the progression of residual hepatocellular carcinoma (HCC) after heat treatment.

**Methods:**

Heat-exposed residual HCC cells were cultured in different ECM gels. We used basement membrane gel (Matrigel) to simulate the normal microenvironment and collagen I to model the pathological stromal ECM. The alterations of morphology and parameters of proliferation, epithelial-mesenchymal transition (EMT) and stemness were analyzed in vitro and in vivo.

**Results:**

Increased collagen I deposition was observed at the periablational zone after incomplete RFA of HCC in a xenograft model. The markers of cell proliferation, EMT, motility and progenitor-like traits of heat-exposed residual HCC cells were significantly induced by collagen I as compared to Matrigel (*p* values all < 0.05). Importantly, collagen I induced the activation of ERK phosphorylation in heat-exposed residual HCC cells. ERK1/2 inhibitor reversed the collagen I-promoted ERK phosphorylation, cell proliferative, protrusive and spindle-like appearance of heat-treated residual HCC cells in vitro. Moreover, collagen I promoted the in vivo tumor progression of heat-exposed residual HCC cells, and sorafenib markedly reversed the collagen I-mediated protumor effects.

**Conclusions:**

Our findings demonstrate that collagen I could enhance the aggressive progression of residual HCC cells after suboptimal heat treatment and sorafenib may be a treatment approach to thwart this process.

**Electronic supplementary material:**

The online version of this article (10.1186/s12885-018-4820-9) contains supplementary material, which is available to authorized users.

## Background

Among the various thermal ablations, radiofrequency ablation (RFA) has gained worldwide use and been deemed as the first-line treatment for unresectable early-stage hepatocellular carcinoma (HCC) with the complete necrosis rate higher than 90% [1–4]. However, using RFA to treat medium-sized or large lesions diminishes the therapeutic efficacy due to the difficulty of achieving sufficient ablative margin, which results in apparent or microscopic residual tumor and a significant increase of local recurrence as high as 60% [5–8]. More importantly, accelerated malignant behaviors induced by insufficient thermal ablation have been increasingly reported [9–11]. However, the mechanism underlying this phenomenon remains unknown.

In the previous studies, sublethal heat treatment induced residual HCC cells themselves displaying more malignant phenotypes [9–11]. Since HCC arises on a background of fibrotic liver, active cross-talk between liver microenvironment and HCC cells (maybe more important) promotes tumor progression [12, 13]. RFA treatment not only destroys the tumors, but also drastically remodels the local tissue microenvironment such as extracellular matrix (ECM) proteins. Besides ECM remodeling, the other factors in post-RFA inflammation reaction also influence the tumor progression after insufficient heat-treatment [14, 15]. However, it attracted our attention that collagen deposit was apparently observed at the perimeter of ablational zone after RFA of heart or liver [16, 17]. Collagen I as one of most abundant ECM proteins has been associated with the increased aggressiveness of many solid tumors including HCC [18–24]. Therefore, it is reasonable to hypothesize that the increased collagen I at periablation stroma would promote the malignant behaviors of residual tumors after insufficient heat treatment.

Here, we presented the importance of collagen I in modulating the progression of residual HCC after heat treatment. Collagen I endowed the heat-exposed residual HCC cells with higher malignancy through the activation of ERK signaling cascade. These unfavorable protumor effects driven by collagen I could be reversed by sorafenib. Our finding helps offer a new treatment strategy to thwart tumor progression of residual HCC after suboptimal RFA.

## Methods

### Cell culture and heat treatment in vitro

Human HCC cell lines MHCC97H (Liver Cancer Institute of Zhongshan Hospital, Fudan University, Shanghai, China) and HepG2 (ATCC, USA) were maintained in DMEM media supplemented with 10% fetal bovine serum (FBS, Gibco) and 1% penicillin/streptomycin in a 5% CO_2_ humidified incubator chamber.

The procedure of in vitro sublethal heat treatment was performed as we previously described [25].

After exposed to sublethal heat treatment, HCC cells were seeded into 6-well plates pre-coated with growth factor-reduced basement membrane gel (Matrigel) (BD, Biosciences) or with gel of collagen I (3 mg/mL, Advanced BioMatrix, San Diego, CA) for desirable incubation periods.

Preparation of collagen I gel was performed according to the manufacturer’s instructions. Briefly, collagen I gels were made by neutralizing rat-tail collagen solution with chilled neutralization solution (Advanced BioMatrix, San Diego, CA) according to the volume ratio of 9:1. The final concentration of collagen I was 3 mg/mL.

### Quantitative reverse transcription-PCR (qRT-PCR)

Briefly, RNA was extracted using TRIZOL reagent (Ambion, CA, USA) and subsequently, cDNA was synthesized and amplified using RevertAid First Strand cDNA synthesis kit and Maxima SYBR Green qPCR Master Mix kit (Thermo Fisher Scientific) according to the manufacturer’s instruction. Primer sequences were presented in Additional file 1: Table S1.

### Western blot

Western blot was carried out as previously described [26]. Total proteins were extracted with lysis buffer (Beyotime Institute of Biotechnology, Shanghai, China) premixed with phenylmethanesulfonyl fluoride (PMSF) and phosphatase inhibitor (Roche). After samples were loaded into gels, electrophoresis, transferring and immunostaining were conducted. The primary antibodies used were: PCNA (1:2000), vimentin (1:1000), E-cadherin (1:1000), N-cadherin (1:1000), Nanog (1:1000), ERK1/2 (1:1000), phosopho-ERK1/2 (Thr202/Tyr204) (1:2000) (Cell Signaling Technology, USA), Tubulin (1:1000) and GAPDH (1:1000) (Beyotime, China). The immunoreactive bands were detected by enhanced chemiluminescence (New cell & Molecular Biotech, China).

### Immunohistochemistry staining

As in the previous description [26], deparaffinization, rehydration, antigen retrieval and immunostaining were performed on formalin-fixed, paraffin-embedded tissue sections. The primary antibodies were: PCNA (1:2000, abcam), Nanog (1:100, CST), E-cadherin (1:100, CST) or phosopho-ERK1/2 (Thr202/Tyr204) (1:200, CST), or collagen I (1:100, Boster). Finally, photographs were captured by the Leica microscope (Germany) with identical settings under high-power magnification (× 200).

### Liver-cell microscopy

As previously described [26], images of cells cultured on the gels of collagen I or Matrigel were dynamically captured every 5 min for 24 h using a Cell-IQ cell culturing platform (Chip-Man Technologies, Tampere, Finland). Cell nucleus was used as the center of point of tracking. At the end of experiments, the track length was depicted by NIH ImageJ software (National Institutes of Health, Bethesda, MD, USA) with the MTrackJ Plugin.

### TCGA analyses

Publically available HCC data (*n* = 374) were downloaded from The Cancer Genome Atlas (TCGA) (http:// cancergenome.nih.gov/) and analyzed using R Statistical Software. The analyses of fragments per kilobase of transcript per million fragments (FPKM) values of genes in TCGA HCC patient samples and survival outcome were conducted as previously described [27].

### Animal experiments

All animal experiments were carried out in compliance with the guidelines by the Shanghai Medical Experimental Animal Care Commission. The experimental protocols were approved by the Ethical Committee on Animal Experiments of Fudan University (Shanghai, China) (Permit Number: 201807002Z).

The MHCC97H orthotopic nude mouse model was developed as previously described [28]. At 4 weeks after orthotopic implantation and tumor size reached about 1.5 cm, mice were subjected to partial RFA or sham ablation. The ablation experiment was performed using Cool-tip RFA system (Covidien, Inc. Boulder, CO, USA) with 1-cm active tip. Briefly, under general anesthesia (pelltobarbitalum natricum, 50 mg/kg, i.p.), the mouse was placed on a conductive metal plate with good electrical conductivity and the tumor was exposed by laparotomy, and a 17-gauge RFA needle was inserted into the center of the tumor. Tumor was ablated at a power output setting of 5 W for 30 s. The RFA generator was not turned on for sham-ablated mouse. After ablation, the skin incision was closed with 5–0 non-absorbable sutures. Mice were euthanized 24 h after ablation and tumor samples were harvested for hematoxylin-eosin staining or Sirius red (Sigma, Israel). Collagen was viewed under bright light using Sirius red staining and collagen I were detected by polarizing light microscopy and immunohistochemistry staining.

Heat-exposed residual HCC cells MHCC97H (2 × 10^7^ cells) suspended in Matrigel alone or together with collagen I solution (1:2) were subcutaneously injected into the upper right flank of mice (4-week-old male BALB/c nude mouse, *n* = 3 for each group). After 2 weeks, animals were sacrificed and tumor samples were collected.

Another 8 mice with the tumors from heat-exposed residual HCC cells MHCC97H (2 × 10^7^ cells) suspended in Matrigel with collagen I solution (1:2) were randomized into two groups: sorafenib group (1.25 mg/kg, i.p., daily for 2-week, *n* = 4) and control group (DMSO, i.p., daily for 2-week, *n* = 4). The tumor weight was estimated following the formula: Tumor weight (mg) = Length(mm) X (Width(mm))^2^/2 [9]. At the end of the experiment (at 2 weeks), tumors samples were harvested for subsequent analyses.

### Statistical analysis

Statistical differences between two groups were calculated using a Student’s two tailed *t-*test. Significance was defined as *P* < 0.05.

## Results

### Collagen I deposit at the periablational zone

**Fig. 1:**
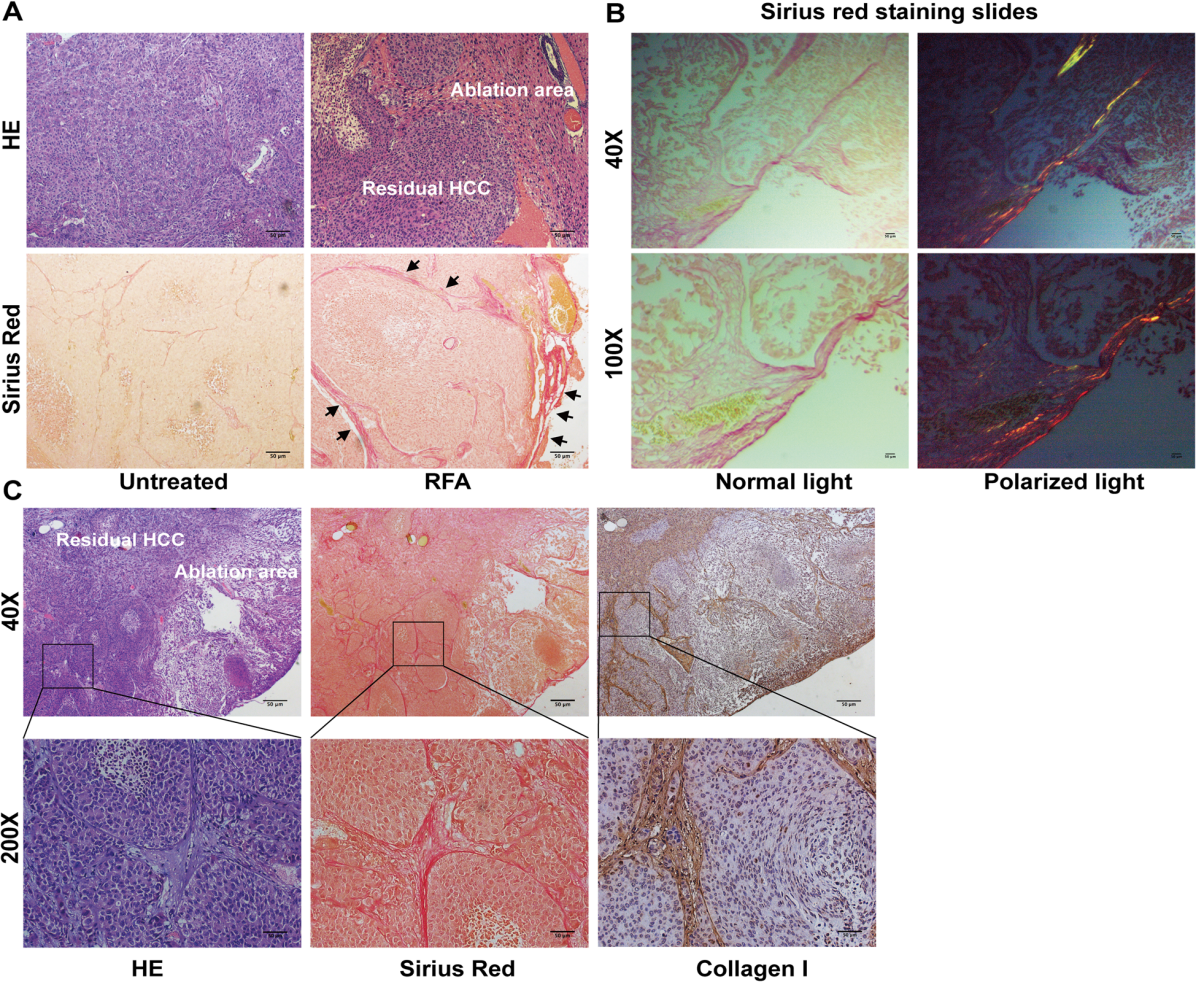
Increased collagen I deposition at the periablational zone after incomplete RFA of HCC in an orthotopic animal model. **a** The increased collagen stained by Sirius Red was located at the periablational zone (black arrow) and surrounded the residual HCC foci. **b** Collagen I deposit (red-yellow color area) was observed using polarized light microphotographs of Sirius red staining-stained slides. **c** The increased collagen I at the periablational zone and around the residual HCC foci was verified by immunohistochemical staining. Scale bar, 50 μm

A HCC orthotopic animal model was subjected to partial RFA. With the use of Sirius red staining, collagen deposit was significantly increased in residual HCC at the periablational zone after incomplete RFA compared with untreated HCC tissues (Fig. 1a). Importantly, in the residual HCC portion, collagen was present around cancer nests. The increased collagen I displayed as red-yellow color area was observed from polarized light microphotographs of Sirius red staining-stained slides (Fig. 1b). Consistently, collagen I deposit was confirmed by immunohistochemistry staining (Fig. 1c). These above results show that collagen I deposit increases at the perimeter of ablated zone and surrounds the residual HCC foci after insufficient RFA, indicating the close relationship between collagen I and residual HCC cells.

### Collagen I promoted the malignant phenotypes of heat-exposed residual HCC cells

**Fig. 2:**
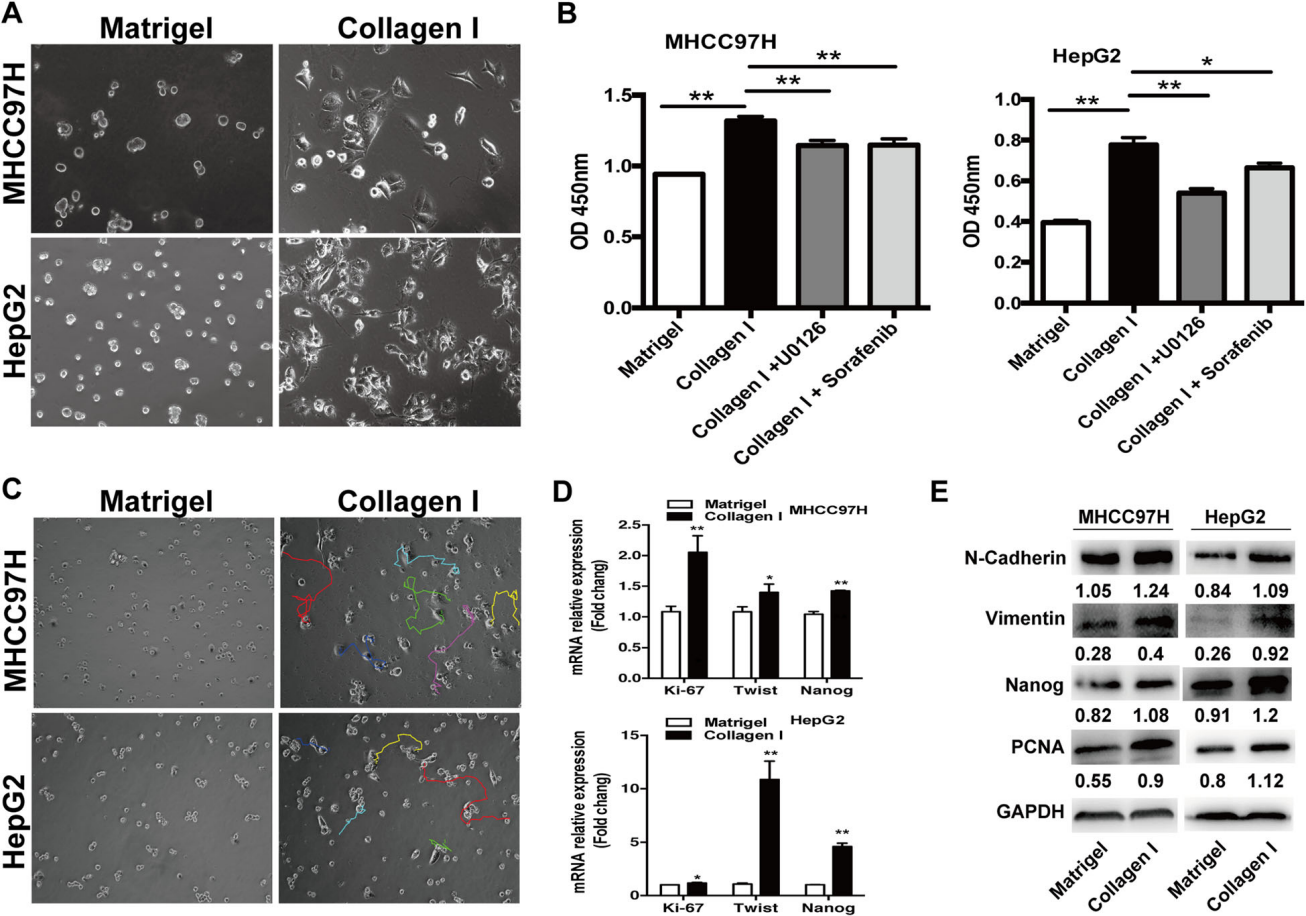
Collagen I stimulated the proliferation, motility, and the expression of EMT and progenitor-like markers in heat-treated residual HCC cells. **a** Compared with the cells cultured on Matrigel, heat-treated residual HCC cells on collagen I displayed a proliferative, protrusive and spindle-like appearance. **b** Collagen I promoted proliferation of heat-treated residual HCC cells as determined by the WST-1 proliferation assay. The OD (optical density) was measured at 450 nm wavelength. **c** Compared with Matrigel, collagen I enhanced the motility of heated-exposed residual HCC cells as demonstrated by tracking analysis. **d** As shown by qRT-PCR, the mRNA expression of Ki-67, twist, and Nanog was increased in heat-exposed residual HCC cells cultured on collagen I versus Matrigel. **e** The increased expression of PCNA, vimentin, N-cadherin and Nanog protein in heat-exposed residual HCC cell cultured on collagen I was detected by Western blot. Expression levels of target proteins were normalized to the corresponding levels of GAPDH. **, *P <* 0.01; *, *P <* 0.05

After exposed to sublethal heat treatment (47 °C for 10 min), HCC cells were seeded on the plates pre-coated with Matrigel gel or collagen I gel. Distinct morphological changes were observed. Heated-exposed residual HCC cells cultured on Matrigel showed less, rounded, and collective growth. In contrast, the cells cultured on collagen I exhibited proliferative, protrusive and spindle-like appearance (Fig. 2a). Collagen I promoted proliferation of heat-treated residual HCC cells as determined by the WST-1 proliferation assay (Fig. 2b). As shown by live cell microscopy, heat-exposed residual HCC cells on collagen I significantly migrated long distances whereas the cells on Matrigel showed slight motility changes, indicating the enhanced motility ability of heat-exposed residual HCC cells response to collagen I in relative to Matrigel (Fig. 2c). Consistent with these observations, the increased gene expression of proliferation marker and EMT parameter (Ki-67 and twist) was shown by qRT-PCR. The up-regulation of PCNA, N-cadherin, vimentin was further confirmed by western blot. Moreover, the expression of progenitor cell marker Nanog mRNA and protein was increased in heat-exposed residual HCC cells on collagen I gels when compared to the cells cultured on Matrigel (Fig. 2d, e). These data demonstrate the importance of collagen I in modulating malignant biological behaviors of heat-exposed residual HCC cells.

### Collagen I mediated aggressiveness via ERK-dependent activation

**Fig. 3:**
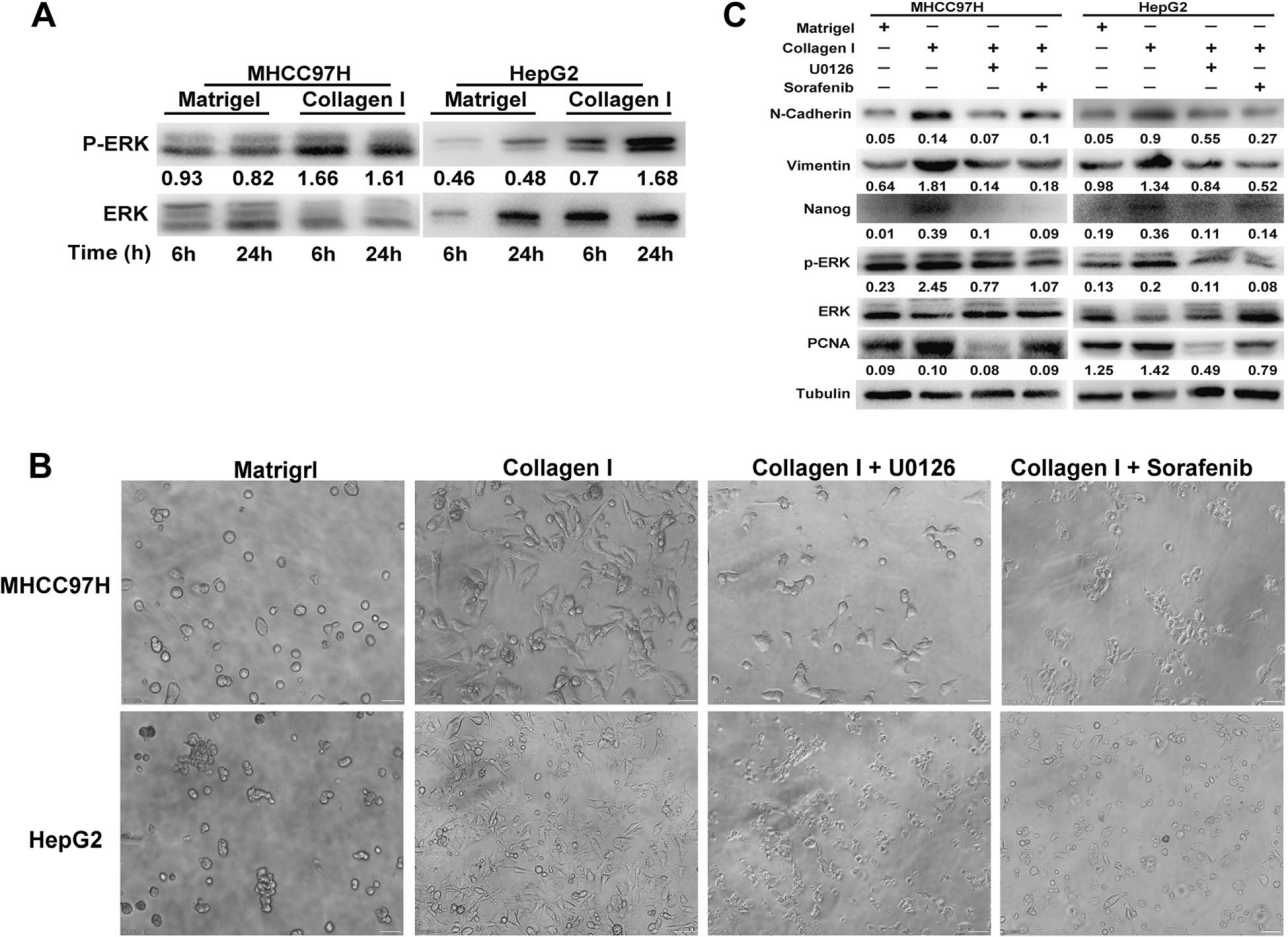
Collagen I induced the activation of ERK in heat-exposed residual HCC cells. **a** The up-regulated level of ERK1/2 phosphorylation were induced in heat-exposed residual HCC cells cultured on collagen I versus Matrigel. The p-ERK content was normalized for ERK. **b** ERK1/2 inhibitor U0126 (25 μM) or sorafenib (5 μM) could reverse the collagen I-promoted proliferative, protrusive and spindle-like appearance of heat-treated residual HCC cells. **c** ERK1/2 inhibitor (U0126, 25 μM) or sorafenib (5 μM) reversed collagen I-mediated upregulation of ERK1/2 in heat-exposed residual HCC cells. Collagen I-induced upregulation of proliferation (PCNA), EMT (vimentin and N-cadherin), cancer stem cell marker Nanog was markedly reduced in heat-exposed residual HCC cells pretreated with ERK1/2 inhibitor (U0126, 25 μM) or sorafenib (5 μM). The p-ERK content was normalized for ERK. Expression levels of PCNA, vimentin, N-cadherin and Nanog were normalized to Tubulin

Activation of ERK has been associated with aggressive phenotypes of HCC [29–31]. Significant upregulation of ERK phosphorylation was observed in heat-exposed residual HCC cells cultured on collagen I versus Matrigel (Fig. 3a). Notably, ERK1/2 inhibitor U0126 could reverse the collagen I-promoted proliferative, protrusive and spindle-like appearance of heat-treated residual HCC cells (Fig. 3b). Consistently, collagen I-induced upregulation of proliferation (PCNA), phosphorylated ERK1/2, EMT (vimentin and N-cadherin), cancer stem cell marker Nanog was markedly reduced in heat-exposed residual HCC cells pretreated with ERK1/2 inhibitor U0126 (Figs. 2b, 3c). These results demonstrate that collagen I inducing the aggressiveness of heat-exposed residual HCC cells is through ERK activation.

### Collagen I promoted the in vivo progression of heat-exposed residual HCC cells

**Fig. 4:**
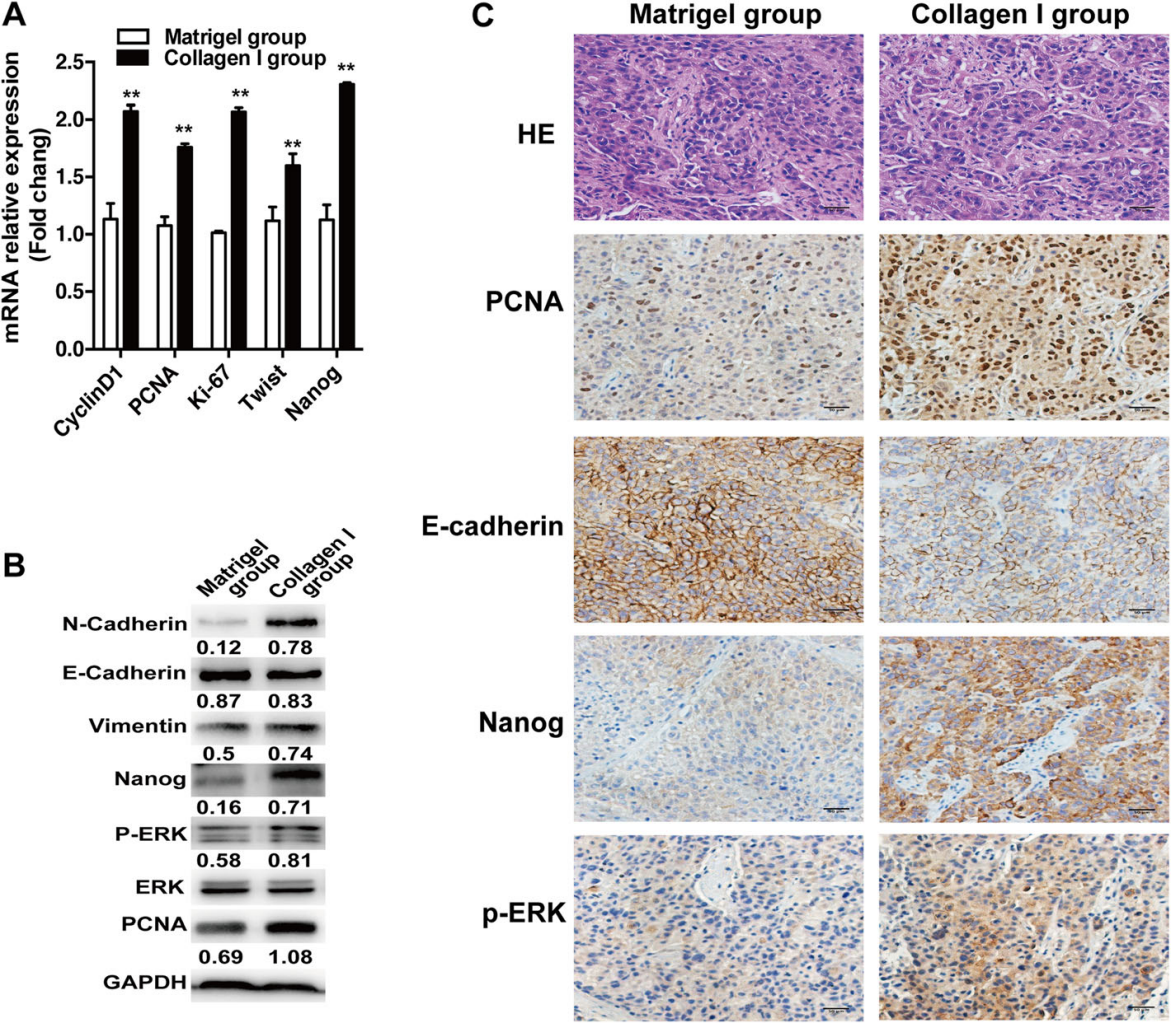
Collagen I promoted the in vivo progression of heat-treated residual HCC cells. **a** The mRNA expression of PCNA, cyclin D1, Ki-67, twist and Nanog was increased in the tumors from heat-exposed residual MHCC97H cells inoculated with collagen I. **b** The protein expression of PCNA, vimentin, N-cadherin, E-cadherin, Nanog and ERK phosphorylation were detected by Western blot. The p-ERK was normalized for ERK. Expression levels of E-cadherin, PCNA, vimentin, N-cadherin and Nanog were normalized to GAPDH. **c** The expression of PCNA, E-Cadherin, Nanog and phosphorylated ERK were evaluated using immunohistochemical staining (scale bar, 50 μm). **, *P <* 0.01; *, *P <* 0.05

To determine whether collagen I would aggravate the in vivo progression of heat-exposed residual HCC, we subcutaneously inoculated heat-treated residual MHCC97H cells mixed with Matrigel alone or together with collagen I into nude mice. Compared with the Matrigel group, tumors of the collagen I group showed the increased expression of proliferation markers (CCND1, PCNA, Ki-67), EMT (twist, vimentin, N-Cadherin), phosphorylated ERK1/2 and Nanog mRNA, and the decreased E-cadherin (Fig. 4a). Consistently, western blot and immunohistochemistry staining analysis showed that the protein levels of PCNA, Nanog, vimentin, N-Cadherin, and phosphorylated ERK1/2 were up-regulated and E-Cadherin was down-regulated in collagen I group (Fig. 4b, c). These results suggest that collagen I could enhance the in vivo aggressiveness of heat-exposed residual HCC cells.

### Sorafenib suppressed the in vivo collagen I-enhanced progression of heat-exposed residual HCC cells

**Fig. 5:**
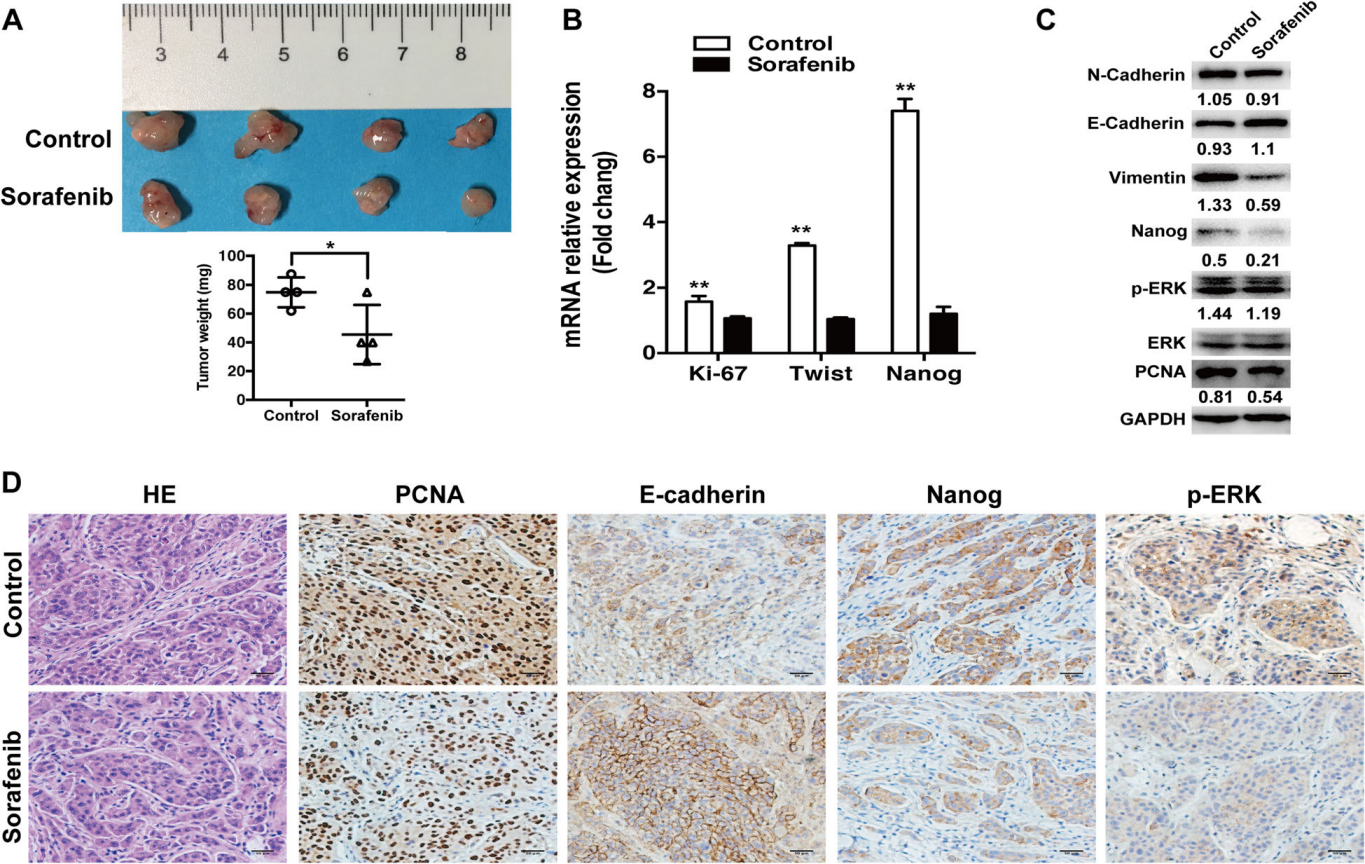
Sorafenib suppressed the in vivo collagen I-induced tumor progression of heat-treated residual HCC cells. **a** Mice with the tumors derived from heat-exposed residual MHCC97H cells with collagen I were subjected to treatment. Compared with the control group, sorafenib significantly inhibited tumor growth. **b** The mRNA expression of Ki-67, twist and Nanog were down-regulated in the sorafenib group. **c** The changes of PCNA, Nanog, vimentin, E-cadherin, N-cadherin, and ERK activation were detected by Western blot. The p-ERK levels were normalized for ERK. Expression levels of E-cadherin, PCNA, vimentin, N-cadherin and Nanog were normalized to GAPDH. **d** The immunohistochemical staining of PCNA, E-Cadherin, Nanog and phosphorylated ERK in the tumors (scale bar, 50 μm). **, *P <* 0.01; *, *P* < 0.05

Sorafenib is the first-line systemic therapy for advanced HCC [32]. To assess whether sorafenib could thwart the collagen I-promoted progression of heat-exposed residual HCC, we used sorafenib to treat the tumors derived from heat-treated residual HCC cells with the mixture of Matrigel and collagen I in nude mice. When compared with the control group, sorafenib significantly reduced tumor growth after 3 weeks (Fig. 5a). Moreover, qRT-PCR and immunoblot showed that sorafenib could markedly reverse the increased expression of proliferation parameters (Ki-67, PCNA) and EMT (twist, vimentin, and N-Cadherin), Nanog, ERK1/2 and the decreased expression of E-cadherin (Fig. 5b, c). These results were also confirmed by immunohistochemistry staining (Fig. 5d). These data indicate that sorafenib could thwart the collagen I-enhanced progression of heat-exposed residual HCC.

### Increased expression of collagen I in HCC tissues

**Fig. 6:**
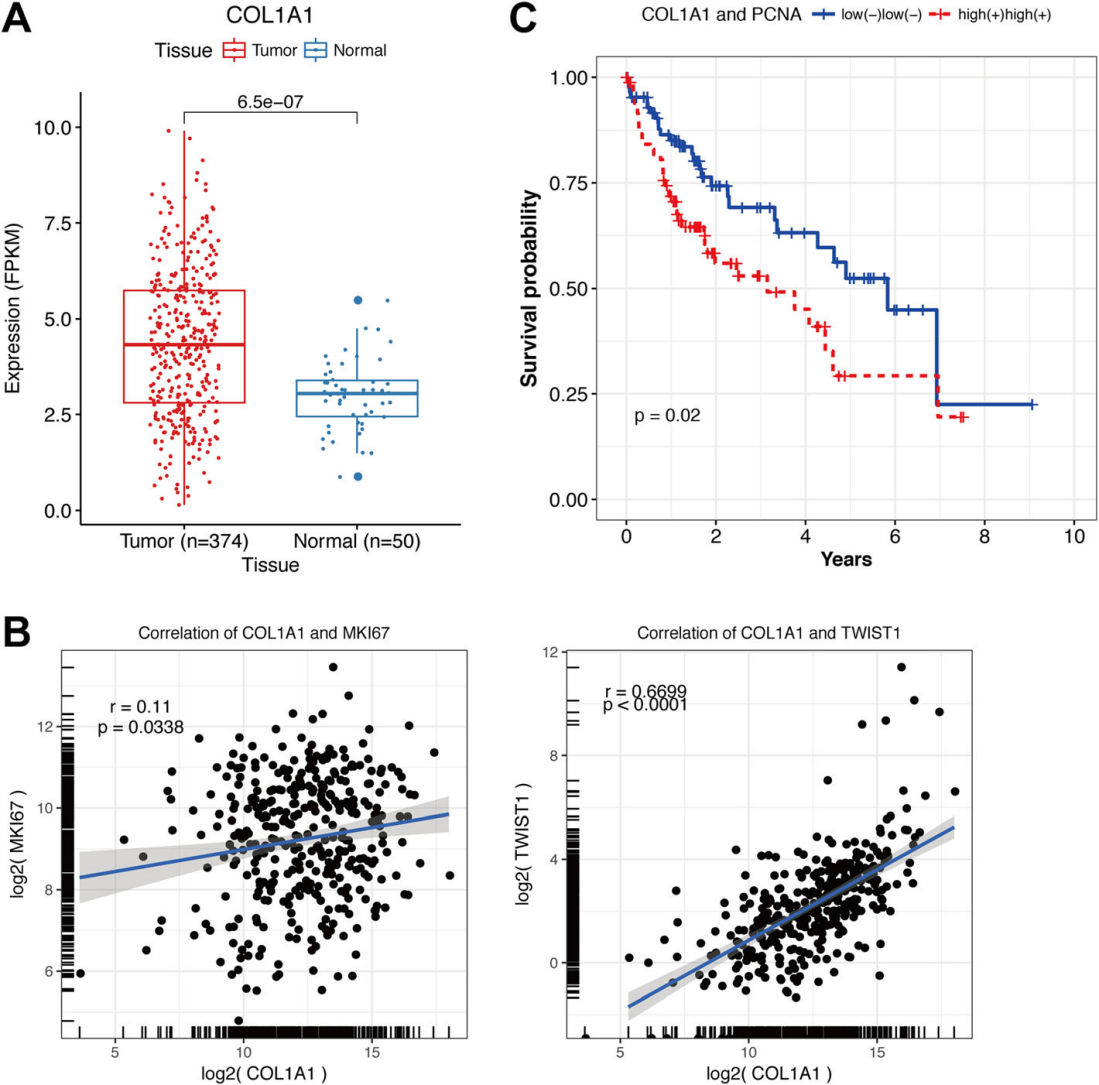
Collagen I expression in TCGA HCC patients. **a** COL1A1 (Collagen I α1 chain) was significantly overexpressed in HCC samples than adjacent non-tumoral tissues. **b** The positive correlations were observed between COL1A1 expression and expression of proliferation marker Ki-67 (*r* = 0.11, *P* = 0.0338) or EMT marker Twist (*r* = 0.6699, *P*<0.0001). **c** High COL1A1 expression combined with expression of PCNA (proliferation marker) predicted unfavorable survival outcomes in TCGA HCC patients

By analyzing data of TCGA HCC cohorts, we found that COL1A1 (collagen I α1 chain) was significantly overexpressed in HCC samples than adjacent non-tumoral tissues (Fig. 6a). The positive correlations were observed between COL1A1 expression and expression of proliferation marker Ki-67 (*r* = 0.11, *P* = 0.0338), or EMT marker Twist (*r* = 0.6699, P<0.0001) (Fig. 6b). In addition, high COL1A1 expression combined with expression of PCNA (proliferation marker) predicted unfavorable survival outcomes in TCGA HCC patients (*p* = 0.02) (Fig. 6c). These results suggest that collagen I expression is associated with cell proliferation and EMT of HCC and predicts poor prognosis of HCC patients.

## Discussion

When RFA is used to treat medium or large HCC, local recurrence and progression occurs frequently due to the remaining of microscopic residual tumors at the periablational zone [33]. Even more, some recurrent HCC after suboptimal RFA could progress rapidly showing the infiltratively growing pattern [34], albeit the mechanism underlying this phenomenon remains unclear. Here, we demonstrate that collagen I, apparently accumulated at the border of ablation zone after RFA, could aggravate the malignant behaviors of heat-treated residual HCC. This unwanted “off-target” effect of thermal ablation provides a new explanation why insufficient RFA could promote the aggressive progression of residual HCC. Second, we propose that sorafenib could reverse this detrimental pro-tumor effect, suggesting a potential treatment approach to thwart residual tumor progression after incomplete RFA. Our findings have clinical implications in improving the therapeutic outcome of RFA.

HCC occurs in the setting of cirrhotic liver with ECM richness [35, 36]. In previous studies, massive collagen deposit was observed at the periphery of periablation zone after RFA of liver tissue [17]. Among the major ECM proteins, the increased collagen I is associated with fibrotic diseases and tumor development [37–40]. Tumor cells or stromal cells (e.g. hepatic stellate cells) may be the source of collagen I [41, 42]. According to literature [42, 43], we assume that collagen I in HCC is mainly produced by activated hepatic stellate cells. After thermal ablation, stromal cells will be recruited around the ablative zone [44] resulting in the increased collagen I deposition at the periablational zone. In the present study, we provided the evidence of the crosstalk between the increased collagen I after RFA and residual HCC cells, that is, collagen I promotes the proliferation, EMT, and progenitor-like characteristics of heat-exposed surviving HCC cells via ERK activation. Also, we show that sorafenib could thwart collagen I-enhanced progression of heat-treated residual HCC cells, indicating a new treatment strategy to inhibit progression of viable HCC after RFA. In line with the previous study, abnormal activation of ERK signaling is correlated with local recurrence of residual HCC after sublethal heat treatment [9, 29–31]. Sorafenib shows survival benefits in advanced HCC patients by blocking many kinase pathways including RAF/MEK/ERK [32, 45–47]. Xu et al. reported that sorafenib inhibited residual HCC progression after incomplete RFA in an animal HCC model [48]. In this study, we further elucidate that sorafenib could block the cross-talk between ECM protein collagen I and residual HCC cells through disrupting ERK signaling.

This study has several limitations. First, besides collagen I, we could not exclude the other factors in post-inflammation reaction after RFA that influence tumor progression of residual HCC [14, 15, 49, 50], such as RFA-induced tumor-specific T-cell reaction, a Th1 cytokine pattern after RFA, heat shock proteins, periablational cellular infiltration. Second, we did not identify the source of collagen I. The thermal ablative environment may stimulate the production of collagen I from tumor cells or stromal cells. Third, we employed a subcutaneous tumor model of implanting heat-treated residual HCC cells in mice to study the response to sorafenib therapy. This model is the absence of liver microenvironment. Better animal models (rabbit VX2 hepatoma, a MDR2-knockout inflammation-induced HCC model resembling human HCC) are needed to verify our findings.

## Conclusions

**Fig. 7:**
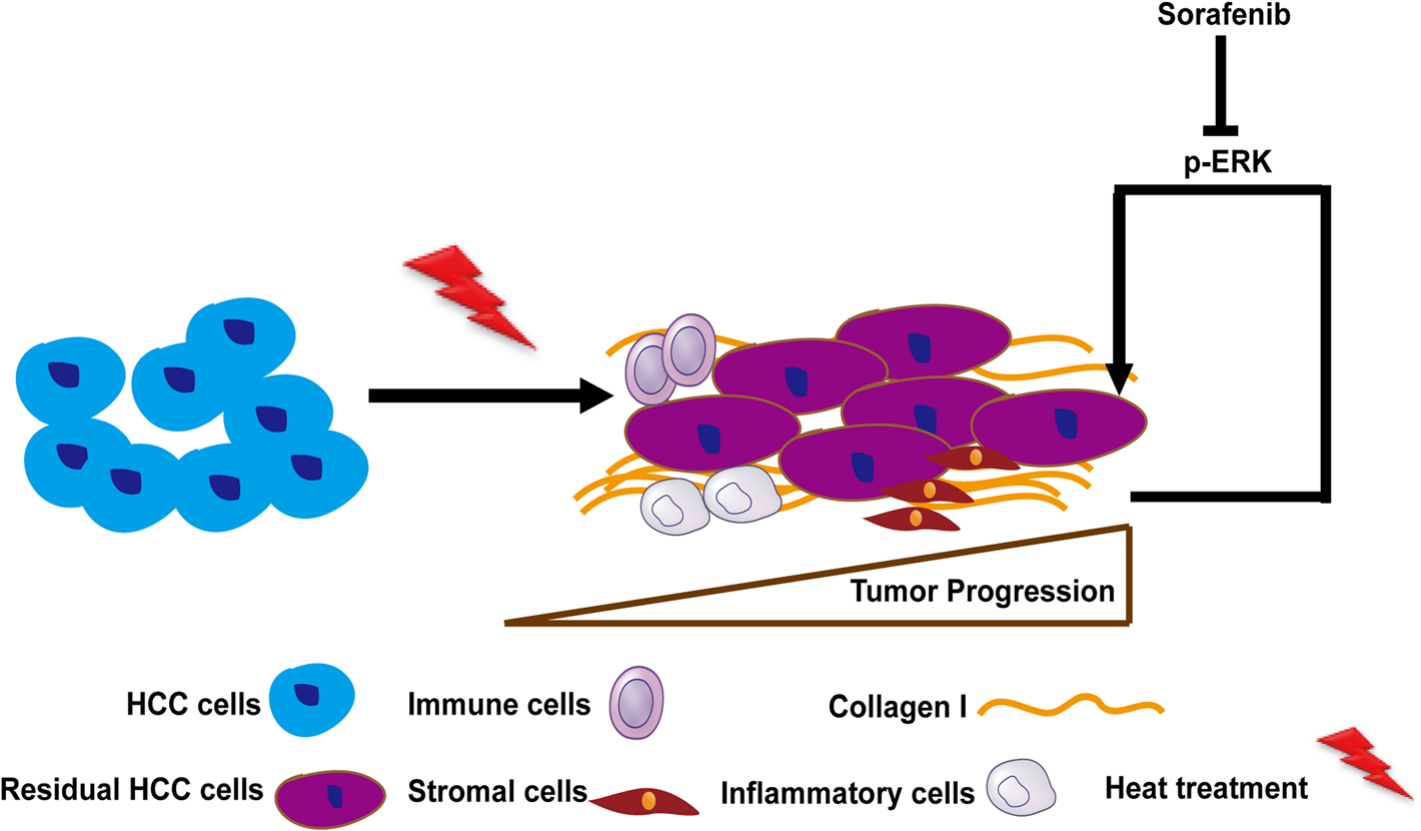
Schematic diagram shows that collagen I initiates ERK signaling to accelerate the aggressive progression of residual HCC cells after sublethal RFA, which could be reversed by sorafenib. More factors implicated in post-inflammation reaction after RFA promote tumor progression of residual HCC, which has been reported by the other authors [14, 15, 49, 50]

In summary (Fig. 7), we reveal that the increased collagen I promotes the progression of heat-exposed residual HCC cells, indicating the importance of the ECM protein collagen I in modulating residual HCC after incomplete heat treatment, and propose that sorafenib could reversed the collagen I-induced protumor effects.

## Additional file

Additional file 1: **Table S1.** Primers for quantitative RT-PCR. (DOCX 13 kb)

## Abbreviations

ECM: Extracellular matrix
EMT: Epithelial-mesenchymal transition
ERK1/2: Extracellular signal-related kinase 1/2
HCC: Hepatocellular carcinoma
RFA: Radiofrequency ablation
