# Supplementary material for: Extracellular matrix collagen I promotes the tumor progression of residual hepatocellular carcinoma after heat treatment
Source: BMC Cancer. 2018 Sep 18;18:901. doi: 10.1186/s12885-018-4820-9 (PMC6145107; doi:10.1186/s12885-018-4820-9)
Supplement: Supplementary file 1 — Table S1. Primers for quantitative RT-PCR. (DOCX 13 kb) [file 12885_2018_4820_MOESM1_ESM.docx]

**Table S1. Primers for quantitative RT-PCR.**

| Gene | Sequence |
| --- | --- |
| PCNA | Forward-5’-ACACTAAGGGCCGAAGATAACG-3’  Reverse-5’-ACAGCATCTCCAATATGGCTGA-3’ |
| Ki-67 | Forward-5’-ACGCCTGGTTACTATCAAAAGG-3’  Reverse-5’-CAGACCCATTTACTTGTGTTGGA-3’ |
| CyclinD1  Twist | Forward-5’- GCTGCGAAGTGGAAACCATC-3’  Reverse-5’- CCTCCTTCTGCACACATTTGAA-3’  Forward-5’-AGCAAGATTCAGACCCTCAAG-3’  Reverse-5’-ATCCTCCAGACCGAGAAGG-3’ |
| NANOG | Forward-5’-AGGCAAACAACCCACTTCTG-3’  Reverse-5’-TCTGCTGGAGGCTGAGGTAT-3’ |
| GAPDH | Forward-5’-GGAGCGAGATCCCTCCAAAAT-3’  Reverse-5’-GGCTGTTGTCATACTTCTCATGG-3’ |
